# Interventional therapy combined with tyrosine kinase inhibitors with or without immune checkpoint inhibitors as initial treatment for hepatocellular carcinoma with portal vein tumor thrombosis: a systematic review and meta-analysis

**DOI:** 10.1007/s12672-024-01026-9

**Published:** 2024-05-14

**Authors:** Changjie Du, Hongyu Wu, Tao Zhong, Qilong Zhai, Jiajun Yuan, Jialun Peng, Rong Ma, Jinzheng Li

**Affiliations:** Department of Hepatobiliary Surgery, The Second Affiliated Hospital of Chongqing Medical University, No.76 Linjiang Road, Yuzhong District, Chongqing, 400010 People’s Republic of China

**Keywords:** Hepatocellular carcinoma, Portal vein tumor thrombosis, Interventional therapy, Tyrosine kinase inhibitors, Immune checkpoint inhibitors

## Abstract

**Background:**

Interventional therapy, in conjunction with tyrosine kinase inhibitors (TKIs), has shown promising outcomes for treating hepatocellular carcinoma (HCC) with portal vein tumor thrombosis (PVTT). With the advent of immunotherapy, the combined use of immune checkpoint inhibitors (ICIs) has attracted great attention due to their potential effectiveness in advanced HCC. This study aims to compare the efficacy and safety of a triple therapy regimen (Interventional therapy, TKIs and ICIs, IT-TKI-ICI) with a dual therapy regimen (Interventional therapy and TKIs, IT-TKI) in the treatment of HCC and PVTT (HCC-PVTT).

**Methods:**

A comprehensive search was carried out in PubMed, Web of Science, Embase, Scopus, and the Cochrane Library databases. Primary outcome measures were overall survival (OS) and progression-free survival (PFS), while secondary outcomes included tumor response rate, adverse event incidence as well as downstaging surgery rate. Statistical analysis was conducted using Revman 5.4 software.

**Results:**

The meta-analysis finally included **6** cohort studies. The triple therapy group demonstrated significantly prolonged OS and PFS compared to the dual therapy group. Meanwhile, the former exhibited significantly higher rates of objective response rate (ORR), disease control rate (DCR) and better downstaging effects with a higher salvage surgery rate without significantly increasing adverse events.

**Conclusion:**

In comparison to dual therapy, the triple therapy with interventional therapy, TKIs, and ICIs demonstrates superior efficacy and equivalent safety for HCC-PVTT.

**Supplementary Information:**

The online version contains supplementary material available at 10.1007/s12672-024-01026-9.

## Introduction

Hepatocellular carcinoma (HCC) is a prevalent malignant tumor of the digestive system, ranking as the third leading cause of cancer-related mortality globally^1^. HCC often infiltrates the portal vein, culminating in the development of portal vein tumor thrombosis (PVTT), Which is a significant factor affecting the prognosis^2,3^. Untreated patients with HCC and PVTT (HCC-PVTT) have a median survival time of only 2.7 months^4^. Currently, there is no international consensus on the treatment of HCC with concurrent PVTT. Guidelines from the European Association for the Study of the Liver advocate systemic treatments like sorafenib, lenvatinib, cabozantinib, or nivolumab^5,6^. In contrast, some experts in Asian countries, including China, Japan, and Korea, propose the use of local interventions such as transarterial chemoembolization (TACE), hepatic arterial infusion chemotherapy (HAIC), portal vein stent and iodine-125 seed strand (PVS-I125) for HCC-PVTT patients to achieve more satisfactory clinical outcomes^7–10^. Retrospective studies suggest that, for patients who suffer from HCC-PVTT, TACE results in a higher tumor response rate and longer median progression-free survival (PFS) and overall survival (OS) compared to monotherapy with anti-angiogenic targeted drugs^11,12^.

However, due to the high malignancy and drug-resistance of hepatocellular carcinoma, standalone approaches often fail to achieve satisfactory clinical results^13^. For unresectable cases, a trend has emerged favoring the combination of local and systemic treatments^13,14^. Local interventional treatments like TACE and HAIC can induce tumor tissue hypoxia or generate inflammatory responses, leading to tumor cell destruction. Because of the subsequent upregulation of pro-angiogenic factors in the tumor tissue post-intervention, the application of tyrosine kinase inhibitors (TKIs) makes it crucial to maximize anti-angiogenic effects^15^. A randomized controlled trial by He et al. reported the efficacy of HAIC in combination with sorafenib compared to sorafenib monotherapy in HCC-PVTT. The combination therapy significantly improved tumor response rates (ORR: 40.8% vs. 2.45%, P < 0.0001) and the survival period of various types of PVTT patients (VP1-2: 18.17 vs. 10.87 months, P = 0.002; VP3: 13.47 vs. 6.27 months, P < 0.001; VP4: 9.47 vs. 5.5 months, P < 0.001)^16^.

Immune checkpoint inhibitors (ICIs), including programmed cell death protein 1 inhibitor (PD-1), programmed cell death ligand 1 inhibitor (PD-L1), and anti-cytotoxic T lymphocyte antigen 4 inhibitor (CTLA-4), have been incorporated into routine treatments for advanced-stage hepatocellular carcinoma^7,14^. ICIs can block the generation of immune tolerance by binding to specific targets on tumor cells or immune cells, allowing the immune cells to re-recognize the tumor^17^. This activation of the host's immune response leads to long-term tumor destruction^18^. There is a potential synergy between ICIs, intervention therapies, and TKIs^15,18,19^. So, several small-sample retrospective studies have compared the efficacy of a triple therapy (Interventional therapy, TKIs and ICIs, IT-TKI-ICI) with a dual therapy (Interventional therapy and TKIs, IT-TKI) for HCC-PVTT, demonstrating extended survival periods for patients undergoing the triple therapy^15,18,20–22^. However, due to the small sample sizes, outcome variations, and a lack of large prospective randomized controlled trials among the currently published studies, there exists inadequate evidence supporting the effectiveness and safety of the triple therapy for patients with HCC-PVTT. Therefore, our goal is to conduct a meta-analysis of existing studies to explore whether the triple therapy, compared to the previous dual therapy, would genuinely bring clinical benefits to patients who suffer from HCC-PVTT.

## Methods

We conducted a meta-analysis of the included studies following the Preferred Reporting Items for Systematic Reviews and Meta-Analyses (PRISMA) guidelines^23^ (www.prisma-statement.org). Since this study is a secondary research with publicly available data, formal approval from an institutional review board or informed consent from patients was not required^24^. The meta-analysis has been registered on PROSPERO (https://www.crd.york.ac.uk/PROSPERO/) with the registration number CRD42023462791.

### Search strategy

We searched databases, including PubMed, Web of Science, EMBASE, Scopus, and the Cochrane Library database, for clinical studies comparing the triple therapy to the dual therapy for HCC-PVTT. The search terms included portal vein tumor thrombosis, interventional therapy, tyrosine kinase inhibitor, immune checkpoint inhibitor, etc. The search period is from the database built until April 24, 2024. Specific terms and keywords are shown in Supplementary Table 1.

### Eligibility criteria

Inclusion Criteria: (1) population: patients with clinical or pathological diagnosis of HCC-PVTT, and no previous relevant local or systemic anti-tumor treatments including interventional therapy, radiotherapy and systemic therapy, but recurrence after single surgery is accepted; (2) intervention: the triple therapy regimen in a combination of IT-TKI-ICI; types of interventional therapy include transarterial chemoembolization (TACE), hepatic arterial infusion chemotherapy (HAIC), portal vein stent and iodine-125 seed strand(PVS-I125), etc.; types of TKIs and ICIs are not restricted; (3) comparison: the dual therapy regimen with IT-TKI; (4) outcome: at least one major outcome indicator and we can directly or indirectly obtain effect measures; major outcome indicators include progression-free survival (PFS) and overall survival (OS); secondary outcome indicators include the number of complete response (CR), partial response (PR), stable disease (SD), progressive disease (PD), overall response rate (ORR), disease control rate (DCR), adverse events (AEs), and downstaging surgery rate (DSR). DSR refers to the proportion of HCC-PVTT patients who can successfully achieve tumor downstaging and undergo salvage surgery after the treatments.

Exclusion Criteria: (1) literature such as reviews, systematic reviews, conference abstracts, comments, letters, editorials, guidelines, animal experiments, etc.; (2) duplicate publications or literature without full-text access; (3) studies that included HCC with and without PVTT, but PVTT data from the two groups could not be separated; (4) literature that cannot directly or indirectly extract outcome indicators.

### Literature screening, data extraction, and quality assessment

After the initial search, two authors (Changjie Du and Jiajun Yuan) independently screened the titles and abstracts of the articles to identify potentially relevant studies. Subsequently, the full-text articles were independently screened and reviewed by the former authors based on the inclusion and exclusion criteria, and literature data as well as quality were extracted. Disagreements were resolved through discussion with a third author (Hongyu Wu) to reach a consensus. Data extraction included the following: author, country, publication date, study design type, basic characteristics of patients (gender, age, liver function, overall condition, etc.), tumor characteristics (tumor size, number of tumors, PVTT classification, distant metastasis), treatment regimens, outcome indicators (PFS, OS, CR, PR, SD, PD, AEs, DSR), etc. For the randomized controlled trial (RCT) data, the Cochrane collaboration tool was used to assess the risk of bias. For non-randomised cohort studies, the Risk Of Bias In Non-randomised Studies—of Interventions (ROBINS-I) tool^25^ and the Newcastle–Ottawa Scale (NOS)^26^ were used to evaluate the quality of included studies. For the results of NOS, a score of 7–9 was considered high-quality research, 4–6 was considered medium-quality research, and less than 4 was considered low-quality research. We included studies of medium to high quality and excluded low-quality studies.

### Data analysis

Data analysis of outcome indicators in the included studies was performed using RevMan 5.4 software. For meta-analysis of tumor response, DSR, and AEs, risk ratios (RRs) were our preferred outcome measure. For meta-analysis of PFS and OS, we preferred hazard ratios (HRs) and mean difference (MD), because HRs can provide time-to-event information and MD can quantify the time of survival. When HRs were not directly available, We would contacted the corresponding authors for them, or we performed secondary data analysis using Kaplan–Meier curves, P values, and median OS and PFS values indirectly^27–29^. Cochrane's Q-test and I^2^ statistics were used to assess heterogeneity^30^. If the P value of Cochrane's Q-test was less than 0.01 and I^2^ statistics were greater than 50%, indicating substantial heterogeneity, a random-effects model would be chosen^31^. Otherwise, heterogeneity was considered acceptable, and a fixed-effects model would be used. Publication bias was assessed using a funnel plot and Egger's test^32^. And the sensitivity analysis was assessed through the meta-analysis ignoring each study in turn^33^. All positive outcomes were re-evaluated using trial sequential analysis (TSA) to further ensure outcome stability and reduce the possibility of false positive results. TSA can provide a threshold for a statistically significant treatment effect, if the cumulative test statistic curve (Z-curve) intersects with the the TSA adjusted significance threshold, it is considered to have a statistical significance and a stable result^34^. If not, it is considered that false positive results may exist, and an extra study population size will be provided to achieve statistical significance.Trial sequential analysis (TSA) was performed with the TSA software (TSA-0.9.5.10-Beta).

## Results

### Retrieval results and study selection

**Fig. 1:**
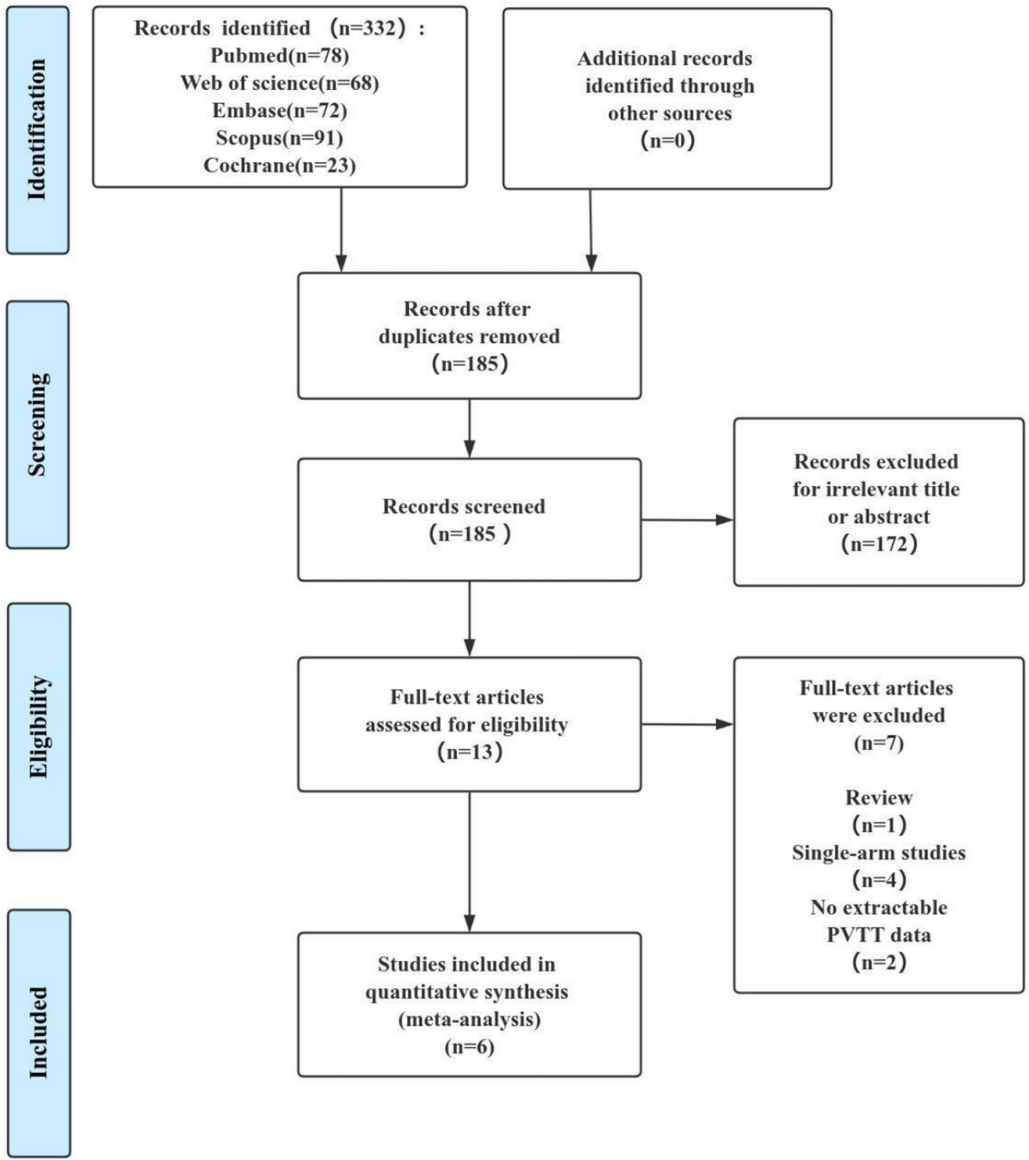
Search strategy for selection of studies

In the initial retrieval strategy, a total of 332 relevant studies have been identified. Following screening based on inclusion and exclusion criteria, as well as quality assessment, we selected 6 retrospective cohort studies for quantitative analysis^15,18,20–22,35^. Moreover, the ROBINS-I tool and the NOS were used to assess the quality of the studies, there was no serious or critical risk of bias observed in ROBINS-I tool (Supplementary Table 2) and all of which were rated as high quality with the NOS (Supplementary Table 3). No studies were excluded from the analysis after quality assessment and ultimately those studies were selected for our meta-analysis. The detailed process is illustrated in Fig. 1.

### Data characteristics, literature situation

Six retrospective studies reported on the comparison between the triple therapy and the dual therapy for patients with HCC-PVTT. As some literature underwent propensity score matching (PSM), we opted for the inclusion of matched cohort data to reduce intergroup differences. A total of 568 patients were included, with 259 (45.6%) receiving the triple therapy and 309 (54.40%) receiving the dual therapy. The included literature was formally published in 2023 and 2024, and the study populations were all Chinese, with sample sizes ranging from 18 to 90 patients. The average age of patients ranged from 47.9 to 60 years, with the majority having a background of hepatitis B, consistent with the Chinese context. All patients had acceptable overall and liver function, theoretically tolerating combined therapy. Detailed characteristics are summarized in Table 1.

**Table 1:** Characteristics of the included studies and the patients

First Author (Year)	Country	Study design (Period)	Quality	Type of PVTT	Treatment	No. of patients	Mean/median age (range)	Male/female	Child–pugh (A/B)	HBV (YES/NO)	AFP (< 400/ ≥ 400)	ECOG 0–1/2	Tumor size	Tumor number	Extra-hepatic metastasis
Lin(2023)	China	Retrospective (2017–2022)	7	VP1-4	TACE + L + P	45	57.0 ± 6.4	42/3	28/17	42/3	24/21	45/0	9.0 ± 3.7	3/42 (< 3/ > 3)	30/15
					TACE + L	50	56.2 ± 11.5	44/6	25/25	43/7	31/19	50/0	8.4 ± 3.1	7/43 (< 3/ > 3)	41/9
Xia(2023)	China	Retrospective (2018–021)	8	Cheng I-III	TACE + A + P	40	29/11 (< 60/ > 60)	36/4	38/2	16/24	24/16	40/0	NA	16/12 (< 3/ > 3)	19/21
					TACE + A	69	47/22 (< 60/ > 60)	61/8	54/15	24/45	35/34	69/0	NA	18/10 (< 3/ > 3)	42/27
Yu(2023)	China	Retrospective (2019–2022)	7	VP3-4	TACE + HAIC + TKI + P	39	50.9 ± 10.9	37/2	27/12	38/1	27/12	36/3	12.4 ± 4.3	16/23 (< 3/ > 3)	27/10
					TACE + HAIC + TKI	37	47.9 ± 11.0	35/2	32/5	36/1	26/11	34/3	11.4 ± 4.1	20/17 (< 3/ > 3)	5/42
Zhang(2023)	China	Retrospective (2018–2021)	8	VP4	TACE + PVS-I125 + L + P	47	23/24 (< 55/ > 55)	42/5	44/3	NA	25/22	45/2	22/25 (> 10/ < 10)	NA	5/42
					TACE + PVS-I125 + L	40	23/17 (< 55/ > 55)	36/4	38/2	NA	29/11	37/3	22/18 (> 10/ < 10)	NA	5/35
Zou(2023)	China	Retrospective (2018–2022)	7	VP2-4	TACE + L + P	70	53.6 ± 15.1	59/11	46/24	63/7	49/21	70/0	57/11 (> 5/ < 5)	36/34 (< 3/ > 3)	48/22
					TACE + L	90	52.3 ± 14.8	77/13	61/29	78/12	63/27	90/0	77/13 (> 5/ < 5)	41/49 (< 3/ > 3)	61/29
Wu(2024)	China	Retrospective (2019–2020)	7	Cheng I-IV	TACE + L + P	18	56.9 ± 8.1	15/3	18/0	16/2	7/11	18/0	16/2 (> 5/ < 5)	2/16 (< 1/ > 1)	4/14
					TACE + L	23	58.1 ± 9.4	18/5	21/2	22/1	13/11	23/0	20/3 (> 5/ < 5)	2/16 (< 1/ > 1)	11/12

*PVTT* portal vein tumor thrombosis, *TACE* transarterial chemoembolization, *HAIC* hepatic arterial infusion chemotherapy, *PVS-I125* portal vein stent and iodine-125 seed, *VP* the Japanese VP classification for PVTT, *Cheng* the Cheng ‘s classification as suggested by Professor Cheng of China for PVTT, *TKI* tyrosine kinase inhibitor, *L* Lenvatinib, *A* apatinib, *P* programmed death-1 inhibitor, *HBV* hepatitis B virus, *AFP* alpha-fetoprotein, *ECOG* Eastern Cooperative Oncology Group, *NA* not available

### Meta-analysis results

We collected 10 measurable outcomes, divided into 3 categories to compare the efficacy and safety of the triple therapy and the dual therapy.

## Tumor response rate and Downstaging Surgery Rate (DSR)

**Fig. 2:**
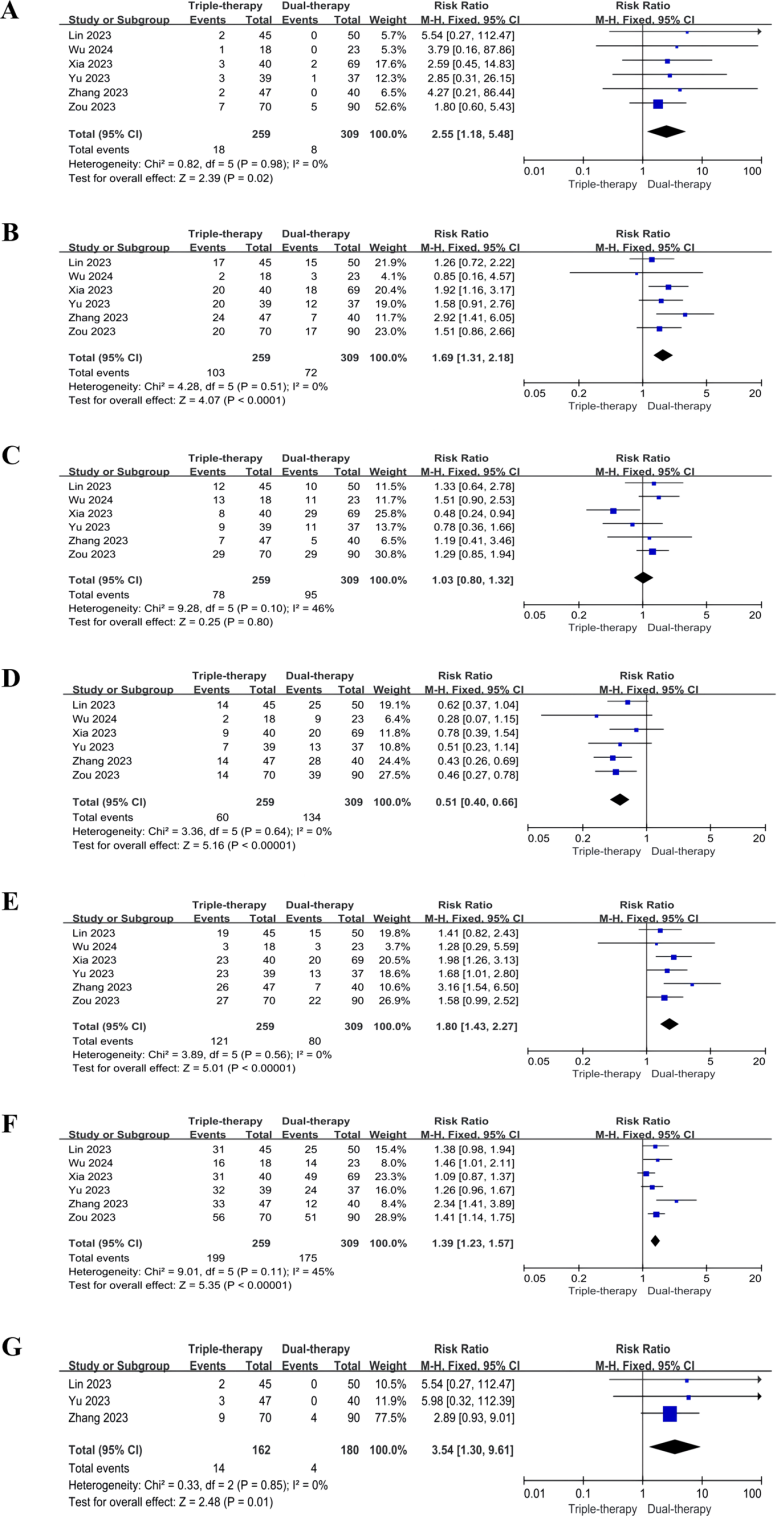
Meta-analysis results of CR (**A**), PR (**B**), SD (**C**), PD (**D**), ORR (**E**), DCR (**F**) and DSR (**G**). *CR* complete response, *PR* partial response, *SD* stable disease, *PD* progressive disease, *ORR* overall response rate, *DCR* disease control rate, *DSR* downstaging surgery rate, *CI* confidence interval

In the comparison of tumor response rates between the triple and dual therapy groups in 6 studies, Cochrane's Q-test for each outcome was greater than 0.1, and all I^2^ statistics were less than 50%, no substantial heterogeneity was found and fixed effect model was adopted. The triple therapy group exhibited significantly higher rates in complete response (CR) rate (RR = 2.55, 95% CI 1.18, 5.48, P = 0.02), partial response (PR) rate (RR = 1.69, 95% CI 1.31, 2.18, P < 0.0001), objective response rate (ORR) (RR = 1.80, 95% CI 1.43, 2.27, P < 0.00001), and disease control rate (DCR) (RR = 1.39, 95% CI 1.23, 1.57, P < 0.00001) when compared to the dual therapy group. Additionally, the triple therapy group had a lower progressive disease (PD) rate (RR = 0.51, 95% CI 0.40, 0.66, P < 0.0001). However, there was no statistically significant difference in stable disease (SD) rate between the two groups (RR = 1.03, 95% CI 0.80, 1.32, P = 0.80). Detailed results are presented in Table 2 and Fig. 2A, B, C, D, E, and F.

**Table 2:** Meta-analysis results of tumor response and survival outcomes

Measured outcomes	No. studies	Total no. Patients (triple/dual)	No. Patients (triple/dual)	Heterogeneity test I2(%)	P	Model	RR/HR/MD	95% CI	P
CR	6	259 vs. 309	18 vs. 8	0	0.98	Fixed	2.55 (RR)	[1.18, 5.48]	0.02
PR	6	259 vs. 309	103 vs. 72	0	0.51	Fixed	1.69 (RR)	[1.31, 2.18]	< 0.0001
SD	6	259 vs. 309	78 vs. 95	46	0.10	Fixed	1.03 (RR)	[0.80, 1.32]	0.80
PD	6	259 vs. 309	60 vs. 134	0	0.64	Fixed	0.51 (RR)	[0.40, 0.66]	< 0.00001
ORR	6	259 vs. 309	121 vs. 80	0	0.56	Fixed	1.80 (RR)	[1.43, 2.27]	< 0.00001
DCR	6	259 vs. 309	183 vs. 161	45	0.11	Fixed	1.39 (RR)	[1.23, 1.57]	< 0.00001
DSR	3	162 vs. 180	14 vs. 4	0	0.85	Fixed	3.54 (RR)	[1.30, 9.61]	0.01
mOS	6	259 vs. 309	247 vs. 268	0	0.50	Fixed	0.63 (HR)	[0.55, 0.73]	< 0.00001
mPFS	6	259 vs. 309	247 vs. 268	0	0.60	Fixed	0.46 (HR)	[0.38, 0.55]	< 0.00001
mOS	4	156 vs. 168	156 vs. 168	0	0.92	Fixed	5.08 (MD)	[2.75, 7.41]	< 0.0001
mPFS	4	156 vs. 168	156 vs. 168	42	0.16	Fixed	3.42 (MD)	[2.32, 4.51]	< 0.00001
VP4 mOS	2	52 vs. 54	52 vs. 54	0	0.38	Fixed	0.65 (HR)	[0.49, 0.85]	0.002
VP4 mPFS	2	52 vs. 54	52 vs. 54	0	0.56	Fixed	0.51 (HR)	[0.35, 0.74]	0.0004
VP4 mOS	2	52 vs. 54	52 vs. 54	0	0.77	Fixed	6.07 (MD)	[3.45, 8.69]	< 0.00001
VP4 mPFS	2	52 vs. 54	52 vs. 54	52	0.15	Fixed	3.16 (MD)	[0.84, 5.48]	0.008

*CR* complete response, *PR* partial response, *SD* stable disease, *PD* progressive disease, *ORR* overall response rate, *DCR* disease control rate, *DSR* downstaging surgery rate, *mOS* median overall survival, *mPFS* median progression-free survival, *P* p-Value, *CI* confidence interval, *RR* risk ratio, *HR* hazard ratio, *MD* mean difference, *VP4* main trunk PVTT (MPVTT)

The DSR was reported in three studies for both the triple therapy and dual therapy groups. No obvious heterogeneity was found in Q-test and I^2^ statistics, and fixed effect model was adopted. The triple therapy group exhibited a higher DSR compared to the latter (RR = 3.54, 95% CI 1.30, 9.61, P = 0.01). Detailed results are shown in Table 2 and Fig. 2G.

## Survival outcome measures (OS and PFS)

**Fig. 3:**
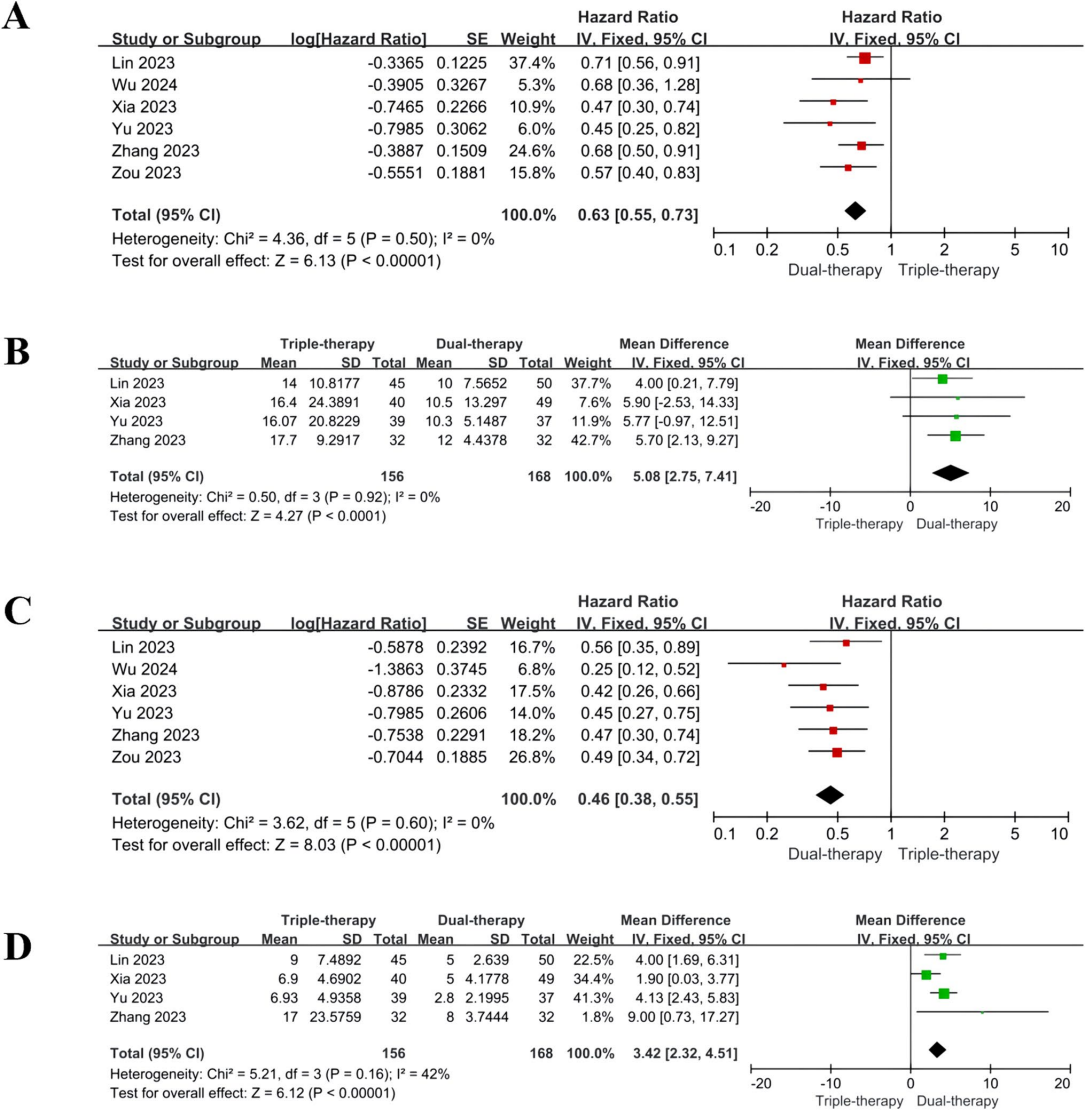
HR results of OS (**A**) and PFS (**C**); MD results of OS (**B**) and PFS (**D**). *OS* overall survival, *PFS* progression-free survival, *CI* confidence interval

Six studies reported survival outcomes, but 3 studies did not provide directly specifying HR values and 95% CI with only median survival times, Kaplan–Meier survival curves and corresponding P values for both OS and PFS. We initially contacted the corresponding authors via email to obtain HR values and 95% CI for OS and PFS and finally achieved it in one study. In the other two studies, we performed secondary data analysis indirectly. No substantial heterogeneity was found in Q-test and I^2^ statistics, and fixed effect model was adopted. Compared to the dual therapy group, the triple therapy group showed significantly higher OS (HR = 0.63, 95% CI 0.55, 0.73, P < 0.00001; MD = 5.08 months, 95% CI: 2.75, 7.41, P < 0.001) and PFS (HR = 0.46, 95% CI 0.38, 0.55, P < 0.0001; MD = 3.42 months, 95% CI 2.32, 4.51, P < 0.001). Detailed results are depicted in Fig. 3A, B, C, and D.

### Subgroup analysis

We stratified the analysis based on PVTT and assessed whether triple therapy had survival benefits with main trunk PVTT (MPVTT).

**Fig. 4:**
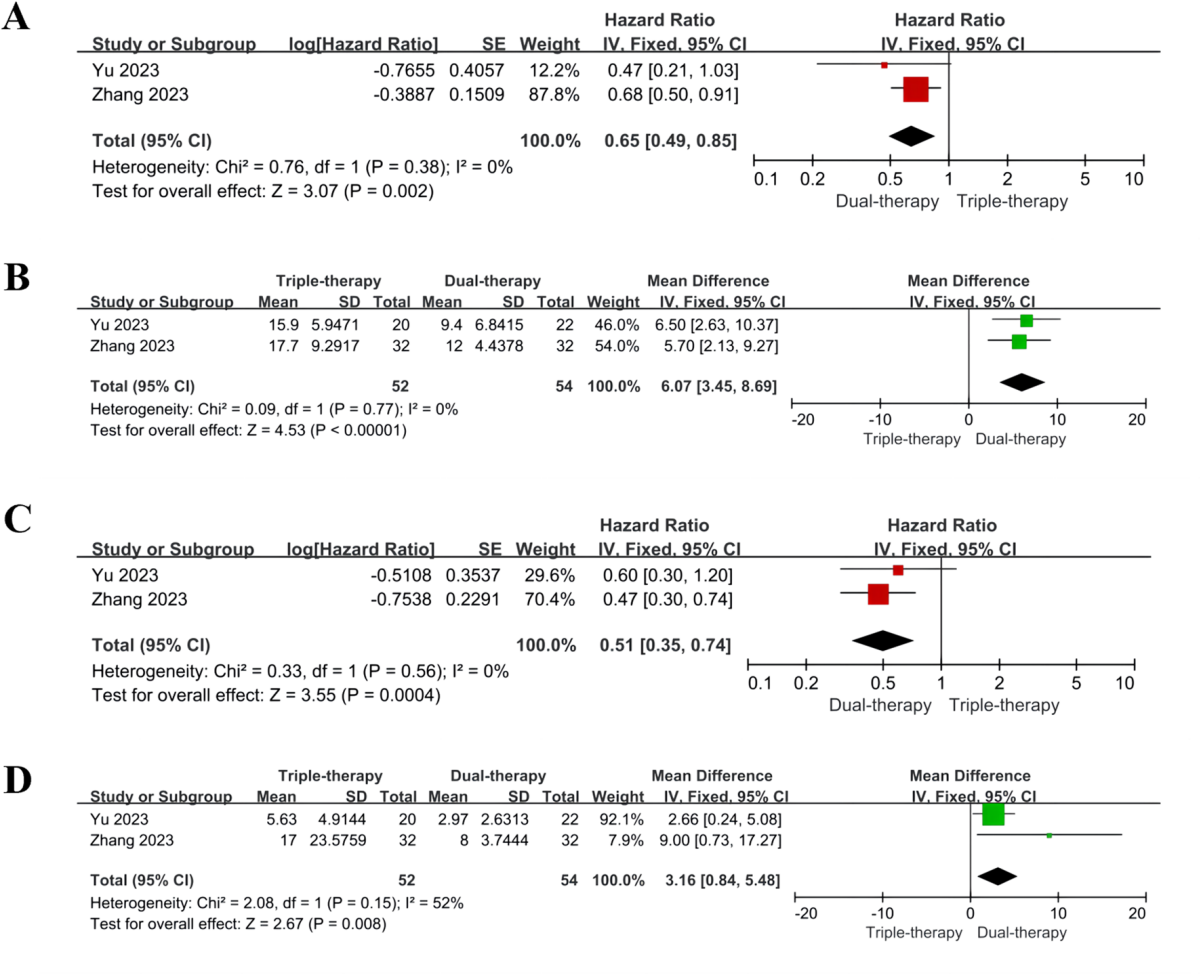
HR results of OS (**A**) and PFS (**C**) for MPVTT; MD results of OS (B) and PFS (D) for MPVTT. *OS* overall survival, *PFS* progression-free survival, *CI* confidence interval, *MPVTT* main trunk portal vein tumor thrombosis

Two studies reported the efficacy of triple therapy in MPVTT, while other studies did not provide subgroup reports based on PVTT subtyping. No substantial heterogeneity was found in Q-test and I^2^ statistics, and fixed effect model was adopted. Compared to the dual therapy group, MPVTT patients in the triple therapy group exhibited significantly increased OS (HR = 0.65, 95% CI 0.49, 0.85, P = 0.002; MD = 6.07 months, 95% CI 3.45, 8.69, P < 0.001) and PFS (HR = 0.51, 95% CI 0.35, 0.74, P = 0.0004; MD = 3.16 months, 95% CI 0.84, 5.48, P = 0.008). Detailed results are depicted in Fig. 4A, B, C, D.

## Adverse events (AEs)

All 6 studies reported AEs for both treatment groups. The total number of AEs in the triple therapy group and dual therapy group was 768 and 839, respectively. The total number of grade 3–4 adverse events was 97 in the triple therapy group and 91 in the dual therapy group. There were no reports of patient deaths due to AEs in either group. Specific and detailed results are depicted in Supplementary Table 4 and Supplementary Table 5.

In terms of IT-TKI treatments, common AEs associated with clinical symptoms and laboratory tests included fever (RR = 0.88, 95% CI 0.63, 1.24, P = 0.46), nausea and vomiting (RR = 1.00, 95% CI 0.73, 1.38, P = 0.98), abdominal pain (RR = 1.02, 95% CI 0.81, 1.28, P = 0.90), fatigue (RR = 1.11, 95% CI 0.84, 1.47, P = 0.46), diarrhea (RR = 1.07, 95% CI 0.70, 1.62, P = 0.77), hyperbilirubinemia (RR = 0.98, 95% CI 0.68, 1.43, P = 0.93), rash (RR = 1.15, 95% CI 0.75, 1.79, P = 0.52), hand-foot syndrome (RR = 1.02, 95% CI 0.77, 1.36, P = 0.88), hypertension (RR = 0.99, 95% CI 0.74, 1.32, P = 0.95), proteinuria (RR = 1.36, 95% CI 0.81, 2.39, P = 0.25), thrombocytopenia (RR = 1.15, 95% CI 0.80, 1.66, P = 0.44), gastrointestinal haemorrhage (RR = 1.18, 95% CI 0.63, 2.19, P = 0.61), oral ulcer (RR = 1.07, 95% CI 0.60, 1.89, P = 0.83) and so on. Detailed results are depicted in Supplementary Table 4. At the same time, specific grade 3–4 AEs were classified separately in Supplementary Table 5, including fatigue (RR = 0.95, 95% CI 0.39, 2.32, P = 0.90), rash (RR = 1.42, 95% CI 0.45, 4.51, P = 0.55), diarrhea (RR = 0.79, 95% CI 0.26, 2.45, P = 0.69), hypertension (RR = 1.05, 95% CI 0.45, 2.45, P = 0.91), hand-foot syndrome (RR = 1.19, 95% CI 0.60, 2.35, P = 0.62), oral ulcer (RR = 1.46, 95% CI 0.46, 4.61, P = 0.52), proteinuria (RR = 0.38, 95% CI 0.06, 2.38, P = 0.30), hyperbilirubinemia (RR = 2.91, 95% CI 0.92, 9.23, P = 0.07), thrombocytopenia (RR = 0.89, 95% CI 0.35, 2.28, P = 0.81) and so on. There were no significant differences between the triple therapy and the dual therapy in all mentioned AEs. Detailed results are depicted in Tables 3 and 4.

**Table 3:** Meta-analysis results of total treatment-related AEs of IT-TKI in two groups

Measured outcomes	No. Studies	Total no. Patients (triple/dual)	Total events (triple/dual)	Heterogeneity test I2(%)	P	Model	RR	95% CI	P
Fever	3	132 vs.159	31 vs.55	0	0.97	Fixed	0.88	[0.63, 1.24]	0.46
Nausea or vomiting	5	220 vs.272	51 vs.65	0	0.90	Fixed	1.00	[0.73, 1.38]	0.98
Abdominal pain	3	132 vs.159	67 vs.77	0	0.63	Fixed	1.02	[0.81, 1.28]	0.90
Fatigue	5	212 vs.269	62 vs.67	0	0.75	Fixed	1.11	[0.84, 1.47]	0.46
Diarrhea	5	219 vs.240	35 vs.37	0	0.71	Fixed	1.07	[0.70, 1.62]	0.77
Hyperbilirubinemia	2	115 vs.140	34 vs.41	0	0.88	Fixed	0.98	[0.68, 1.43]	0.93
Rash	3	155 vs.209	30 vs.32	0	0.42	Fixed	1.15	[0.75, 1.79]	0.52
Hand-foot syndrome	6	259 vs.309	61 vs.77	0	0.54	Fixed	1.02	[0.77, 1.36]	0.88
Hypertension	6	259 vs.309	61 vs.77	0	0.90	Fixed	0.99	[0.74, 1.32]	0.95
Proteinuria	5	212 vs.269	25 vs.22	0	0.94	Fixed	1.36	[0.81, 2.29]	0.25
Thrombocytopenia	4	172 vs.200	44 vs.43	0	0.76	Fixed	1.15	[0.80, 1.66]	0.44
Gastrointestinal haemorrhage	4	142 vs.179	17 vs.17	0	0.49	Fixed	1.18	[0.63, 2.19]	0.61
Liver abscess	2	84 vs.87	1 vs.2	24	0.25	Fixed	0.69	[0.11, 4.22]	0.69
Digestive ulcer	2	86 vs.77	3 vs.0	0	0.78	Fixed	3.63	[0.43, 31.53]	0.24
Gingival bleeding	2	84 vs.87	5 vs.8	0	0.96	Fixed	0.65	[0.25, 1.92]	0.44
Oral ulcer	3	149 vs.196	18 vs.22	0	0.95	Fixed	1.07	[0.60, 1.89]	0.83
Hoarseness	3	128 vs.182	8 vs.15	0	0.60	Fixed	0.75	[0.33, 1.70]	0.50
Albumin decreased	2	63 vs.73	16 vs.13	0	0.78	Fixed	1.65	[0.68, 3.56]	0.29
New ascites	2	63 vs.73	24 vs.28	0	0.35	Fixed	0.95	[0.46, 1.95]	0.89
Elevated serum AST or ALT	2	63 vs.73	54 vs.62	0	0.91	Fixed	1.04	[0.40, 2.27]	0.94
Decreased appetite	2	63 vs.73	20 vs.17	0	0.53	Fixed	1.51	[0.70, 3.26]	0.29
Leukocytopenia	3	102 vs.110	24 vs.17	21	0.28	Fixed	1.66	[0.83, 3.33]	0.15

*P* p-Value, *CI* confidence interval, *RR* risk ratio, *AEs* adverse events, *IT-TKI* Interventional therapy and TKIs

**Table 4:** Meta-analysis results of grade 3–4 AEs of IT-TKI in two groups

Measured outcomes	No. Studies	Total No. Patients (triple/dual)	Total events (triple/dual)	Heterogeneity test I2(%)	P	Model	RR	95% CI	P
Fatigue	3	154 vs.177	8 vs.10	0	0.82	Fixed	0.95	[0.39, 2.32]	0.90
Rash	2	115 vs.140	6 vs.5	0	0.64	Fixed	1.42	[0.45, 4.51]	0.55
Diarrhea	3	156 vs.167	4 vs.6	0	0.48	Fixed	0.79	[0.26, 2.45]	0.69
Hypertension	6	259 vs.309	9 vs.10	0	0.97	Fixed	1.05	[0.45, 2.45]	0.91
Hand-foot syndrome	4	194 vs.246	15 vs.15	0	0.67	Fixed	1.19	[0.60, 2.35]	0.62
Oral ulcer	2	109 vs.127	6 vs.5	0	0.73	Fixed	1.46	[0.46, 4.61]	0.52
Proteinuria	3	127 vs.150	0 vs.3	0	0.99	Fixed	0.38	[0.06, 2.38]	0.30
Hyperbilirubinemia	2	115 vs.140	10 vs.4	0	0.40	Fixed	2.91	[0.92, 9.23]	0.07
Thrombocytopenia	3	127 vs.150	7 vs.9	0	0.78	Fixed	0.89	[0.35, 2.28]	0.81
Gastrointestinal haemorrhage	2	57 vs.60	3 vs.1	39	0.20	Fixed	2.21	[0.38, 12.87]	0.38
Elevated serum AST or ALT	2	63 vs.73	4 vs.4	0	0.52	Fixed	1.24	[0.29, 5.31]	0.78

*P* p-Value, *CI* confidence interval, *RR* risk ratio, *AEs* adverse events, *IT-TKI* Interventional therapy and TKIs

Additionally, in the triple therapy group, 4 studies (n = 144) separately reported adverse events caused by ICIs (n = 26, 18.06%), with the most common being immune-related thyroid dysfunction (n = 14, 9.72%) and immune-related pneumonia (n = 3, 2.08%). The occurrence of grade 3–4 immune-related adverse events was less frequent (n = 6, 4.17%).

## Publication bias assessment and sensitivity analysis

**Fig. 5:**
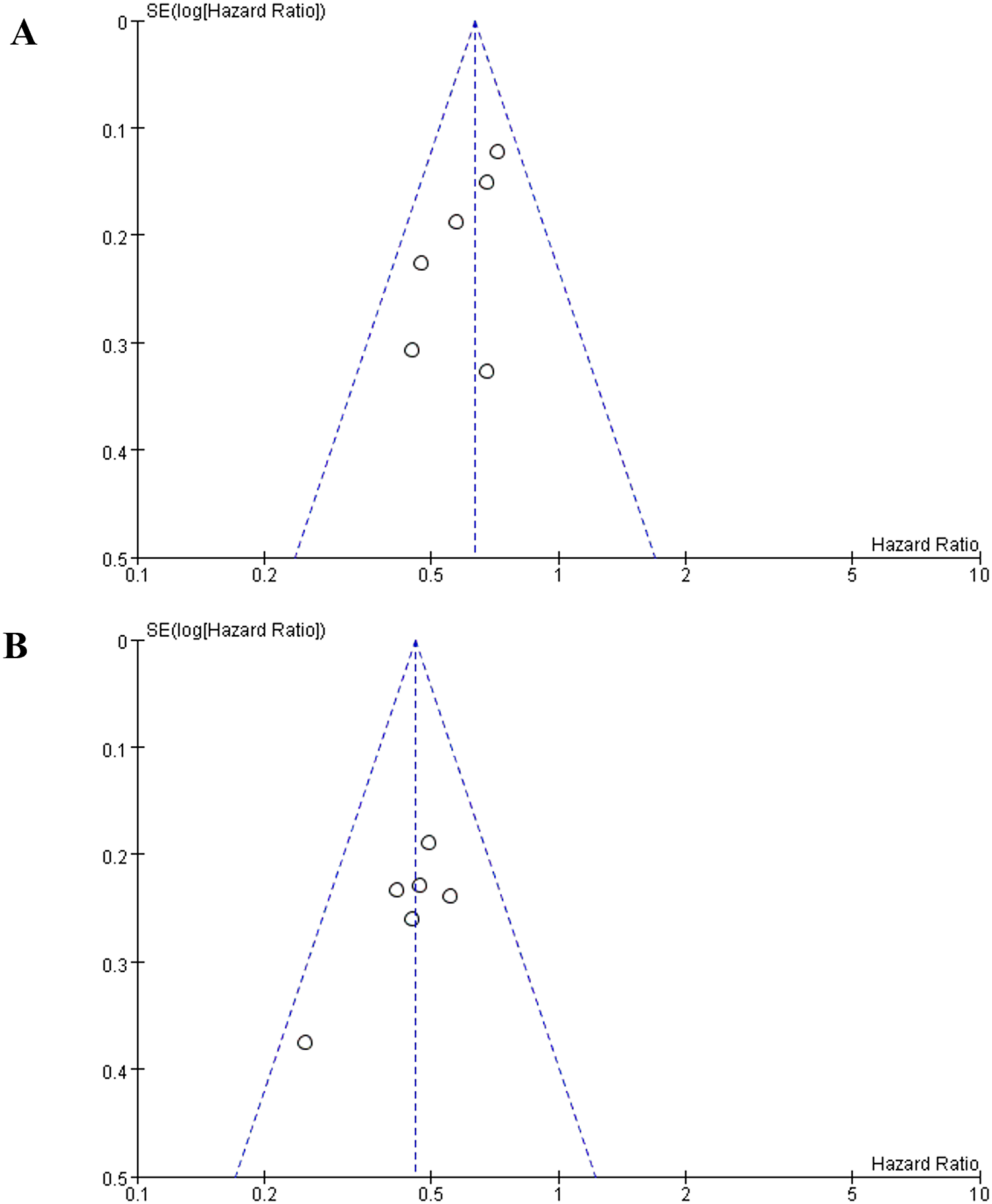
Funnel plot results of OS (**A**) and PFS (**B**). *OS* overall survival, *PFS* progression-free survival

Publication bias was assessed for OS and PFS. All funnel plots are symmetric and all P value of Egger's test were more than 0.05 (OS: P = 0.115; PFS: P = 0.058), which means there is no significant publication bias. Detailed results are depicted in Fig. 5. And the sensitivity analysis showed no significant change by ignoring each study in turn in each meta-analysis, indicating that the results of our meta-analysis were stable.

## Trial sequential analysis (TSA)

**Fig. 6:**
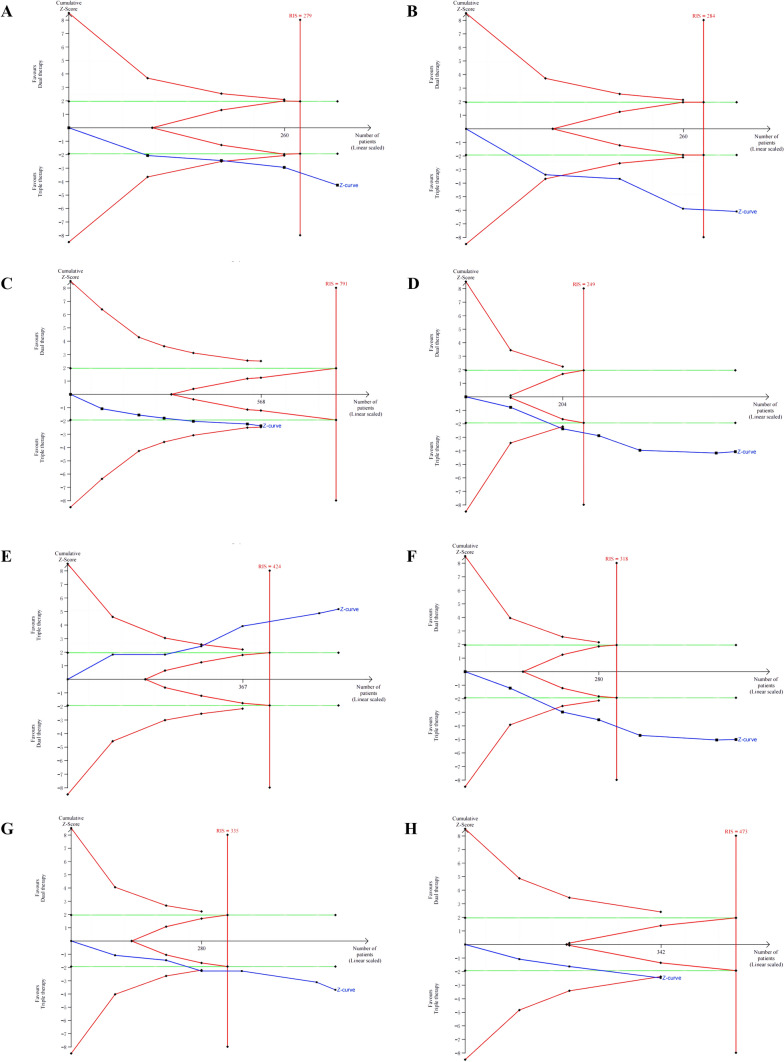
Trial sequential analysis for OS (**A**), PFS (**B**), CR (**C**), PR (**D**), PD (**E**), ORR (**F**), DCR (**G**), and DSR (**H**). *RIS* required information size

Trial sequential analysis was assessed for mean difference (MD) of OS and PFS and relative risk (RR) of CR, PR, PD, ORR, DCR, and DSR. The TSA results were shown in Fig. 6. The results showed that the Z-curves of OS (6A), PFS (6B), PR (6D), PD (6E), ORR (6F), DCR (6G) and DSR (6H) were all intersected with the TSA adjusted significance threshold, which means a statistical significance and a stable result. The Z-curve of CR (6C) exceeded the traditional significance value, but did not intersect with the TSA adjusted significance threshold, suggesting that there was a possibility of false positive outcome, and another study with a total sample of 322 cases may be needed to achieve a statistically significant difference.

## Discussion

The probability of PVTT in advanced-stage HCC ranges from 44.0 to 62.2%^36^. This significantly impacts hepatic blood perfusion, especially when combined with main trunk PVTT (MPVTT), leading to aggravated portal hypertension, further deterioration of liver function, and an increased risk of tumor dissemination^15^. And it is a crucial factor contributing to poor prognosis^37^. However, the optimal treatment for HCC combined with PVTT remains controversial. In recent years, interventional therapy based on TACE has gradually matured, gaining widespread recognition for its efficacy^7,8^. Consequently, in clinical practice, there have been efforts to explore the possibility of combining local treatments like TACE with different systemic treatment regimens to extend the survival of patients with HCC-PVTT^38–41^.

Multiple studies have reported that the combination therapy such as TACE with tyrosine kinase inhibitors (TKIs) which yields higher ORR and DCR compared to the use of TACE or TKIs alone. This combination has also significantly prolonged PFS and OS^42–45^. Additionally, recent studies have investigated a novel triple therapy combining ICIs with interventional therapy and TKIs for the treatment of HCC-PVTT^20–22,46^. Compared to the dual therapy, it remains further comprehensive studies to analyze whether this new approach can provide higher clinical benefits and acceptability.

This study included six retrospective studies involving 568 HCC patients with PVTT^15,18,20–22,35^. In terms of effectiveness, we evaluated survival period, tumor response, and downstaging surgery rate. The triple therapy group demonstrated a longer OS compared with the dual therapy group (HR = 0.63, 95% CI 0.55, 0.73, P < 0.00001; MD = 5.08 months, 95% CI 2.75, 7.41, P < 0.001). Considering that adjustments or changes in the treatment plan may be required following tumor progression, PFS may more accurately reflect the efficacy of a treatment strategy. Therefore, we simultaneously assessed the PFS, which was also significantly prolonged in the triple therapy group (HR = 0.46, 95% CI 0.38, 0.55, P < 0.0001; MD = 3.42 months, 95% CI 2.32, 4.51, P < 0.001). Consistent with these findings, the triple therapy group exhibited higher ORR (RR = 1.80, 95% CI 1.43, 2.27, P < 0.00001) and DCR (RR = 1.39, 95% CI 1.23, 1.57, P < 0.00001) than the dual therapy group. The corresponding CR and PR were 2.55 times (P = 0.02) and 1.69 times (P < 0.001) higher, and the SD and PD were 1.03 times (P = 0.80) and 0.51 times (P < 0.00001), respectively. Moreover, a higher number of patients (n = 11) in the triple therapy group achieved tumor downstaging and underwent salvage liver resection during the treatment, indicating a potential conversion strategy for advanced HCC.

Meanwhile, we conducted a subgroup analysis for HCC patients with MPVTT. Yu et al.^18^ and Zhang et al.^22^ added ICIs to the basis of TKIs with TACE and HIAC or TACE and PVS-I125, respectively, and compared its efficacy with the original regimen for MPVTT patients. Our findings show that the inclusion of ICIs benefits MPVTT patients and leads to a significant extension of OS (HR = 0.65, 95% CI 0.49, 0.85, P = 0.002; MD = 6.07 months, 95% CI 3.45, 8.69, P < 0.001) and PFS (HR = 0.51, 95% CI 0.35, 0.74, P = 0.0004; MD = 3.16 months, 95% CI 0.84, 5.48, P = 0.008). Both HAIC and PVS-I125 may produce potential synergistic effects with ICIs. Previous studies have shown that HAIC achieves high concentration of chemotherapy drugs in a short time to kill tumor cells for tumor antigen release and change in the proportion of immune cells, which can achieve to activate the body's immune system^47,48^. And PVS-I125 not only emits radiation to reduce the tumor load and enhances the invasion and localization of immune cells to the tumor, but also leads portal vein recanalization, increases blood supply and reduces the risk of TACE causing liver failure^49,50^. For MPVTT patients, the IT-TKI-ICI triple regimen based on TACE combining with HAIC or TACE combining with PVS-I125 is full of potential. However, more basic and clinical trials are needed to explore the causal relationship and potential mechanisms between interventional therapy and ICIs in the future. Additionally, due to variations in the specific clinical drugs and applications of TKIs and ICIs, we were unable to extract corresponding patient characteristics and survival outcomes from the included studies. The efficacy and safety of specific TKIs and ICIs regimens cannot be analyzed or recommended at this time.

Finally, we conducted a comprehensive analysis of the adverse reaction rates related to IT-TKI in both groups. Relevant adverse events included fever, abdominal pain, nausea and vomiting, diarrhea, fatigue, hand-foot syndrome, hypertension, proteinuria and so on. No significant differences were observed between the two groups in the incidence of total or grade 3–4 AEs. This is consistent with previous reports on triple therapy in the treatment of advanced HCC, suggesting that the addition of ICIs did not significantly increase the incidence of AEs originally associated with the combination of IT-TKI. Furthermore, the incidence of AEs induced by ICIs itself was relatively low, indicating the relative safety of the triple therapy approach for patients with HCC-PVTT. These results of efficacy and safety demonstrate that the combination of IT-TKI-ICI may be a highly promising comprehensive treatment strategy for patients with HCC-PVTT.

Our analysis has several limitations. Firstly, all eligible studies were retrospective, introducing the risk of selection bias. Secondly, all studies originated from China, and the results of this meta-analysis may not apply to patients in other countries. Thirdly, some survival data in our analysis were obtained through secondary analysis, which may introduce some bias compared to the original data. Lastly, our meta-analysis did not delve into the specific efficacy and safety of TKIs and ICIs drugs. We plan to continuously update our meta-analysis as further research becomes available.

## Conclusion

Compared with dual therapy (IT-TKI), the triple therapy (IT-TKI-ICI) not only markedly increases the local tumor response rate and downstaging surgery rate but also leads to a substantial improvement in long-term survival. Furthermore, the incorporation of ICIs does not result in a notable increase in adverse events compared to the dual therapy. Nevertheless, it is imperative to conduct further prospective, multicenter studies to meticulously evaluate the long-term efficacy and safety of this triple therapy approach. Additionally, we advocate for stratified analyses based on different PVTT classifications to determine the optimal therapeutic strategies to tailor for specific PVTT patient subsets.

### Supplementary Information

Supplementary material 1.

## Data Availability

All data we used in this work can be found in the references.

## Abbreviations

HCC: Hepatocellular carcinoma
PVTT: Portal vein tumor thrombosis
MPVTT: Main trunk portal vein tumor thrombosis
HCC-PVTT: HCC and PVTT
TKIs: Tyrosine kinase inhibitors
ICIs: Immune checkpoint inhibitors
IT-TKI-ICI: Interventional therapy, TKIs and ICIs
IT-TKI: Interventional therapy and TKIs
TACE: Transarterial chemoembolization
HAIC: Hepatic arterial infusion chemotherapy
PVS-I125: Portal vein stent and iodine-125 seed strand
PD-1: Programmed cell death protein 1
PD-L1: Programmed cell death ligand 1
CTLA-4: Anti-cytotoxic T lymphocyte antigen 4 inhibitor
PRISMA: The Preferred Reporting Items for Systematic Reviews and Meta-Analyses
OS: Overall survival
PFS: Progression-free survival
mOS: Median overall survival
mPFS: Median progression-free survival
CR: Complete response
PR: Partial response
SD: Stable disease
PD: Progressive disease
ORR: Objective response rate
DCR: Disease control rate
DSR: Downstaging surgery rate
AEs: Adverse events
RCT: Randomized controlled trial
NOS: The Newcastle–Ottawa Scale
RRs: Risk ratios
MD: Mean difference
HRs: Hazard ratios
PSM: Propensity score matching
TSA: Trial sequential analysis
CI: Confidence interval
L: Lenvatinib
A: Apatinib
HBV: Hepatitis B virus
AFP: Alpha-fetoprotein
ECOG: Eastern Cooperative Oncology Group
NA: Not available
