# Supplementary material for: Interventional therapy combined with tyrosine kinase inhibitors with or without immune checkpoint inhibitors as initial treatment for hepatocellular carcinoma with portal vein tumor thrombosis: a systematic review and meta-analysis
Source: Discov Oncol. 2024 May 14;15:164. doi: 10.1007/s12672-024-01026-9 (PMC11093946; doi:10.1007/s12672-024-01026-9)
Supplement: Supplementary file 1 — Supplementary material 1. [file 12672_2024_1026_MOESM1_ESM.docx]

| **Supplementary table 1**  **The retrieval strategy in our meta-analysis** | | |
| --- | --- | --- |
| **Database** | **Step** | **Strategy** |
| **Pubmed** | #1 | ((((portal vein tumor) OR (portal vein tumor thromb*)) AND (((((((interventional therapy) OR (TACE)) OR (transarterial Chemoembolization)) OR (HAIC)) OR (hepatic arterial infusion chemotherapy)) OR (iodine-125)) OR (endovascular brachytherapy))) AND (((((((tyrosine kinase inhibitor) OR (antiangiogenic-targeted therapy)) OR (Sorafenib)) OR (Lenvatinib)) OR (Apatinib)) OR (Donafenib)) OR (regorafenib))) AND ((((((((((((((((((((PD-1) OR (immune checkpoint inhibitor)) OR (anti-PD-1 antibodies)) OR (anti-PD-1 antibody)) OR (PD-1 inhibitor)) OR (programmed death-1 inhibitor)) OR (Programmed Cell Death Protein 1 Inhibitor)) OR (PD 1 Inhibitor)) OR (PD L1 Inhibitor)) OR (Immune Checkpoint Inhibition)) OR (Immune Checkpoint Blockade)) OR (Camrelizumab)) OR (Toripalimab)) OR (Tislelizumab)) OR (Pembrolizumab)) OR (Sintilimab)) OR (Nivolumab)) OR (Atezolizumab)) OR (Durvalumab)) OR (Envafolimab)) |
| **Web of science** | #1 | (TS=(portal vein invasion)) OR TS=(portal vein tumor thromb*) |
|  | #2 | ((((((TS=(interventional therapy)) OR TS=(TACE)) OR TS=(transarterial Chemoembolization)) OR TS=(HAIC)) OR TS=(hepatic arterial infusion chemotherapy)) OR TS=(iodine-125)) OR TS=(endovascular brachytherapy) |
|  | #3 | ((((((TS=(tyrosine kinase inhibitor)) OR TS=(antiangiogenic-targeted therapy)) OR TS=(Sorafenib)) OR TS=(Lenvatinib)) OR TS=(Apatinib)) OR TS=(Donafenib)) OR TS=(regorafenib) |
|  | #4 | (((((((((((((((((((TS=(PD-1)) OR TS=(immune checkpoint inhibitor)) OR TS=(anti-PD-1 antibodies)) OR TS=(anti-PD-1 antibody)) OR TS=(PD-1 inhibitor)) OR TS=(programmed death-1 inhibitor)) OR TS=(Programmed Cell Death Protein 1 Inhibitor)) OR TS=(PD 1 Inhibitor)) OR TS=(PD L1 Inhibitor)) OR TS=(Immune Checkpoint Inhibition)) OR TS=(Immune Checkpoint Blockade)) OR TS=(Camrelizumab)) OR TS=(Toripalimab)) OR TS=(Tislelizumab)) OR TS=(Pembrolizumab)) OR TS=(Sintilimab)) OR TS=(Nivolumab)) OR TS=(Atezolizumab)) OR TS=(Durvalumab)) OR TS=(Envafolimab) |
|  | #5 | #1 AND #2 AND #3 AND #4 |
| **Embase** | #1 | portal vein invasion:ab,kw,ti |
|  | #2 | portal vein tumor thromb*:ab,kw,ti |
|  | #3 | #1 OR #2 |
|  | #4 | interventional therapy:ab,kw,ti |
|  | #5 | TACE:ab,kw,ti |
|  | #6 | transarterial Chemoembolization:ab,kw,ti |
|  | #7 | HAIC:ab,kw,ti |
|  | #8 | hepatic arterial infusion chemotherapy:ab,kw,ti |
|  | #9 | iodine-125:ab,kw,ti |
|  | #10 | endovascular brachytherapy:ab,kw,ti |
|  | #11 | #4 OR #5 OR #6 OR #7 OR #8 OR #9 OR #10 |
|  | #12 | tyrosine kinase inhibitor:ab,kw,ti |
|  | #13 | antiangiogenic-targeted therapy:ab,kw,ti |
|  | #14 | Sorafenib:ab,kw,ti |
|  | #15 | Lenvatinib:ab,kw,ti |
|  | #16 | Apatinib:ab,kw,ti |
|  | #17 | Donafenib:ab,kw,ti |
|  | #18 | regorafenib:ab,kw,ti |
|  | #19 | #12 OR #13 OR #14 OR #15 OR #16 OR #17 OR #18 |
|  | #20 | PD-1:ab,kw,ti |
|  | #21 | immune checkpoint inhibitor:ab,kw,ti |
|  | #22 | anti-PD-1 antibodies:ab,kw,ti |
|  | #23 | anti-PD-1 antibody:ab,kw,ti |
|  | #24 | PD-1 inhibitor:ab,kw,ti |
|  | #25 | programmed death-1 inhibitor:ab,kw,ti |
|  | #26 | Programmed Cell Death Protein 1 Inhibitor:ab,kw,ti |
|  | #27 | PD 1 Inhibitor:ab,kw,ti |
|  | #28 | PD L1 Inhibitor:ab,kw,ti |
|  | #29 | Immune Checkpoint Inhibition:ab,kw,ti |
|  | #30 | Immune Checkpoint Blockade:ab,kw,ti |
|  | #31 | Camrelizumab:ab,kw,ti |
|  | #32 | Toripalimab:ab,kw,ti |
|  | #33 | Tislelizumab:ab,kw,ti |
|  | #34 | Pembrolizumab:ab,kw,ti |
|  | #35 | Sintilimab:ab,kw,ti |
|  | #36 | Nivolumab:ab,kw,ti |
|  | #37 | Atezolizumab:ab,kw,ti |
|  | #38 | Durvalumab:ab,kw,ti |
|  | #39 | Envafolimab:ab,kw,ti |
|  | #40 | #20 OR #21 OR #22 OR #23 OR #24 OR #25 OR #26 OR #27 OR #28 OR #29 OR #30 OR #31 OR #32 OR #33 OR #34 OR #35 OR #36 OR #37 OR #38 OR #39 |
|  | #41 | #3 AND #11 AND #19 AND #40 |
| **Scopus** | #1 | ( ( TITLE-ABS-KEY ( portal  vein  tumor  thromb* ) ) OR ( TITLE-ABS-KEY ( portal  vein invasion ) ) ) AND ( ( TITLE-ABS-KEY ( interventional  therapy ) ) OR ( TITLE-ABS-KEY ( tace ) ) OR ( TITLE-ABS-KEY ( transarterial  chemoembolization ) ) OR ( TITLE-ABS-KEY ( haic ) ) OR ( TITLE-ABS-KEY ( hepatic  arterial  infusion  chemotherapy ) ) OR ( TITLE-ABS-KEY ( iodine-125 ) ) OR ( TITLE-ABS-KEY ( endovascular  brachytherapy ) ) ) AND ( ( TITLE-ABS-KEY ( tyrosine  kinase  inhibitor ) ) OR ( TITLE-ABS-KEY ( antiangiogenic-targeted  therapy ) ) OR ( TITLE-ABS-KEY ( sorafenib ) ) OR ( TITLE-ABS-KEY ( lenvatinib ) ) OR ( TITLE-ABS-KEY ( apatinib ) ) OR ( TITLE-ABS-KEY ( donafenib ) ) OR ( TITLE-ABS-KEY ( regorafenib ) ) ) AND ( ( TITLE-ABS-KEY ( pd-1 ) ) OR ( TITLE-ABS-KEY ( immune  checkpoint  inhibitor ) ) OR ( TITLE-ABS-KEY ( anti-pd-1  antibodies ) ) OR ( TITLE-ABS-KEY ( pd-1  inhibitor ) ) OR ( TITLE-ABS-KEY ( programmed  death-1  inhibitor ) ) OR ( TITLE-ABS-KEY ( anti-pd-1  antibody ) ) OR ( TITLE-ABS-KEY ( programmed  cell  death  protein 1 inhibitor ) ) OR ( TITLE-ABS-KEY ( pd 1 inhibitor ) ) OR ( TITLE-ABS-KEY ( pd  l1  inhibitor ) ) OR ( TITLE-ABS-KEY ( immune  checkpoint  inhibition ) ) OR ( TITLE-ABS-KEY ( camrelizumab ) ) OR ( TITLE-ABS-KEY ( toripalimab ) ) OR ( TITLE-ABS-KEY ( tislelizumab ) ) OR ( TITLE-ABS-KEY ( pembrolizumab ) ) OR ( TITLE-ABS-KEY ( sintilimab ) ) OR ( TITLE-ABS-KEY ( nivolumab ) ) OR ( TITLE-ABS-KEY ( atezolizumab ) ) OR ( TITLE-ABS-KEY ( durvalumab ) ) OR ( TITLE-ABS-KEY ( envafolimab ) ) ) |
| **Cochrane** | #1 | portal vein invasion |
|  | #2 | portal vein tumor thromb* |
|  | #3 | #1 OR #2 |
|  | #4 | interventional therapy |
|  | #5 | TACE:ab,kw,ti |
|  | #6 | transarterial Chemoembolization |
|  | #7 | HAIC:ab,kw,ti |
|  | #8 | hepatic arterial infusion chemotherapy |
|  | #9 | iodine-125 |
|  | #10 | endovascular brachytherapy |
|  | #11 | #4 OR #5 OR #6 OR #7 OR #8 OR #9 OR #10 |
|  | #12 | tyrosine kinase inhibitor |
|  | #13 | antiangiogenic-targeted therapy |
|  | #14 | Sorafenib |
|  | #15 | Lenvatinib |
|  | #16 | Apatinib |
|  | #17 | Donafenib |
|  | #18 | regorafenib |
|  | #19 | #12 OR #13 OR #14 OR #15 OR #16 OR #17 OR #18 |
|  | #20 | PD-1 |
|  | #21 | immune checkpoint inhibitor |
|  | #22 | anti-PD-1 antibodies |
|  | #23 | anti-PD-1 antibody |
|  | #24 | PD-1 inhibitor |
|  | #25 | programmed death-1 inhibitor |
|  | #26 | Programmed Cell Death Protein 1 Inhibitor |
|  | #27 | PD 1 Inhibitor |
|  | #28 | PD L1 Inhibitor |
|  | #29 | Immune Checkpoint Inhibition |
|  | #30 | Immune Checkpoint Blockade |
|  | #31 | Camrelizumab |
|  | #32 | Toripalimab |
|  | #33 | Tislelizumab |
|  | #34 | Pembrolizumab |
|  | #35 | Sintilimab |
|  | #36 | Nivolumab |
|  | #37 | Atezolizumab |
|  | #38 | Durvalumab |
|  | #39 | Envafolimab |
|  | #40 | #20 OR #21 OR #22 OR #23 OR #24 OR #25 OR #26 OR #27 OR #28 OR #29 OR #30 OR #31 OR #32 OR #33 OR #34 OR #35 OR #36 OR #37 OR #38 OR #39 |
|  | #41 | #3 AND #11 AND #19 AND #40 |
| TS: searches title, abstract and indexing; TITLE: title; ABS: abstract; KEY: keywords; ab: abstract; ti: article title; kw: keywords. | | |

| **Supplementary Table 2**  **Risk of bias of included studies according to ROBINS-I tool** | | | | | | | | |
| --- | --- | --- | --- | --- | --- | --- | --- | --- |
| **Study** | **Confounding** | **Selection of participants into study** | **Classification of intervention** | **Deviations from intended interventions** | **Missing data** | **Measurement of outcomes** | **Selection of reported results** | **Results** |
| **Lin（2023）** | Low | Low | Low | Moderate | Low | Low | Low | Moderate |
| **Xia（2023）** | Low | Low | Low | Moderate | Low | Low | Low | Moderate |
| **Yu（2023）** | Low | Low | Low | Moderate | Low | Low | Low | Moderate |
| **Zhang（2023）** | Low | Low | Low | Moderate | Low | Low | Low | Moderate |
| **Zou（2023）** | Low | Low | Low | Moderate | Low | Low | Low | Moderate |
| **Wu（2024）** | Low | Low | Low | Moderate | Low | Low | Low | Moderate |

| **Supplementary table 3**  **Quality assessment of included studies** | | | | | | | | | | | |
| --- | --- | --- | --- | --- | --- | --- | --- | --- | --- | --- | --- |
| **First Author (Year)** | **Study**  **Design** | **Selection** | | | | **Comparability** | | **Outcome** | | | **Score** |
|  |  | 1* | 2* | 3* | 4* | 5* | 6* | 7* | 8* | 9* |  |
| **Lin（2023）** | R | * | * | * | * | * |  | * | * |  | 7 |
| **Xia（2023）** | R | * | * | * | * | * | * | * | * |  | 8 |
| **Yu（2023）** | R | * | * | * | * | * |  | * | * |  | 7 |
| **Zhang（2023）** | R | * | * | * | * | * | * | * | * |  | 8 |
| **Zou（2023）** | R | * | * | * | * | * |  | * | * |  | 7 |
| R: Retrospective comparative study; 1*. Representativeness of exposed cohort; 2*. Selection of non-exposed cohort; 3*. Ascertainment of exposure; 4*. Outcomeof interest was not present at start of study; 5*. Study controls for gender, age, liver function, overall condition and tumor characteristics; 6*. Study controls for any additional factors; 7*. Assessment of outcomes; 8*. Was follow-up long enough for outcomes to occur; 9*. Adequacy of follow-up; R: Retrospective comparative study; PSM: Propensity score matching. | | | | | | | | | | | |

| **Supplementary Table 4**  **Total AEs and grade 3-4 adverse events of IT-TKI in two groups** | | | | | | | | | | | | | | | | | | | | | | | | |
| --- | --- | --- | --- | --- | --- | --- | --- | --- | --- | --- | --- | --- | --- | --- | --- | --- | --- | --- | --- | --- | --- | --- | --- | --- |
|  | **Lin 2023** | | | | **Xia 2023** | | | | **Yu 2023** | | | | **Zhang 2023** | | | | **Zou 2023** | | | | **Wu 2024** | | | |
|  | **Triple** | | **Dual** | | **Triple** | | **Dual** | | **Triple** | | **Dual** | | **Triple** | | **Dual** | | **Triple** | | **Dual** | | **Triple** | | **Dual** | |
|  | **Total** | **G3-4** | **Total** | **G3-4** | **Total** | **G3-4** | **Total** | **G3-4** | **Total** | **G3-4** | **Total** | **G3-4** | **Total** | **G3-4** | **Total** | **G3-4** | **Total** | **G3-4** | **Total** | **G3-4** | **Total** | **G3-4** | **Total** | **G3-4** |
| **Fever** | 9 | 0 | 12 | 0 | 17 | 0 | 32 | 2 | * | * | * | * | 11 | 0 | 11 | 0 | * | * | * | * | * | * | * | * |
| **Nausea or vomiting** | 2 | 0 | 3 | 0 | 13 | 0 | 21 | 0 | * | * | * | * | 14 | 0 | 9 | 0 | 19 | 2 | 27 | 3 | 3 | 0 | 5 | 0 |
| **Abdominal pain** | 23 | 0 | 25 | 0 | 19 | 1 | 28 | 6 | * | * | * | * | 25 | 0 | 24 | 0 | * | * | * | * | * | * | * | * |
| **Fatigue** | 24 | 2 | 24 | 2 | 3 | 0 | 5 | 0 | 8 | 1 | 5 | 2 | * | * | * | * | 20 | 5 | 28 | 6 | 7 | 0 | 5 | 0 |
| **Diarrhea** | 12 | 0 | 16 | 0 | * | * | * | * | 5 | 0 | 2 | 2 | 4 | 1 | 4 | 0 | 11 | 3 | 13 | 4 | 3 | 0 | 2 | 0 |
| **Hyperbilirubinemia** | 19 | 3 | 22 | 2 | * | * | * | * | * | * | * | * | * | * | * | * | 15 | 7 | 19 | 2 | * | * | * | * |
| **Rash** | 13 | 3 | 14 | 3 | 0 | 0 | 3 | 0 | * | * | * | * | * | * | * | * | 17 | 3 | 15 | 2 | * | * | * | * |
| **Hand-foot syndrome** | 18 | 4 | 12 | 3 | 16 | 2 | 31 | 2 | 10 | 3 | 11 | 5 | 2 | 0 | 3 | 0 | 14 | 6 | 17 | 5 | 1 | 0 | 3 | 0 |
| **Hypertension** | 14 | 2 | 16 | 2 | 14 | 1 | 20 | 1 | 9 | 2 | 7 | 2 | 4 | 0 | 6 | 1 | 18 | 3 | 25 | 3 | 2 | 1 | 3 | 1 |
| **Proteinuria** | 14 | 0 | 11 | 0 | 1 | 0 | 2 | 0 | 1 | 0 | 1 | 1 | * | * | * | * | 7 | 0 | 5 | 1 | 2 | 0 | 3 | 1 |
| **Thrombocytopenia** | 12 | 0 | 14 | 0 | * | * | * | * | 14 | 4 | 11 | 3 | * | * | * | * | 12 | 3 | 14 | 5 | 6 | 0 | 4 | 1 |
| **Gastrointestinal haemorrhage** | 10 | 0 | 11 | 0 | 2 | 0 | 4 | 0 | 5 | 3 | 1 | 0 | * | * | * | * | * | * | * | * | 0 | 0 | 1 | 1 |
| **Liver abscess** | 0 | 0 | 2 | 0 | * | * | * | * | 1 | 1 | 0 | 0 | * | * | * | * | * | * | * | * | * | * | * | * |
| **Digestive ulcer** | * | * | * | * | * | * | * | * | 2 | 0 | 0 | 0 | 1 | 0 | 0 | 0 | * | * | * | * | * | * | * | * |
| **Gingival bleeding** | 3 | * | 5 | * | * | * | * | * | 2 | 0 | 3 | 0 | * | * | * | * | * | * | * | * | * | * | * | * |
| **Oral ulcer** | * | * | * | * | 2 | 0 | 4 | 0 | 2 | 1 | 2 | 1 | * | * | * | * | 14 | 5 | 16 | 4 | * | * | * | * |
| **Hoarseness** | * | * | * | * | 1 | 0 | 5 | 0 | * | * | * | * | * | * | * | * | 7 | 1 | 9 | 2 | 0 | 0 | 1 | 0 |
| **Albumin decreased** | 12 | * | 10 | * | * | * | * | * | * | * | * | * | * | * | * | * | * | * | * | * | 4 | 0 | 3 | 0 |
| **New ascites** | 20 | * | 25 | * | * | * | * | * | * | * | * | * | * | * | * | * | * | * | * | * | 4 | 0 | 3 | 0 |
| **Elevated serum AST or ALT** | 40 | 2 | 44 | 1 | * | * | * | * | * | * | * | * | * | * | * | * | * | * | * | * | 14 | 2 | 18 | 3 |
| **Decreased appetite** | 17 | * | 13 | * | * | * | * | * | * | * | * | * | * | * | * | * | * | * | * | * | 3 | 0 | 4 | 0 |
| **Leukocytopenia** | 5 | * | 7 | * | * | * | * | * | 14 | 3 | 6 | 1 | * | * | * | * | * | * | * | * | 5 | 1 | 4 | 0 |
| **Creatinine increased** | 2 | * | 0 | * | * | * | * | * | * | * | * | * | * | * | * | * | * | * | * | * | * | * | * | * |
| **Cholesteatoma** | 1 | * | 0 | * | * | * | * | * | * | * | * | * | * | * | * | * | * | * | * | * | * | * | * | * |
| **Cholecystitis** | 2 | * | 0 | * | * | * | * | * | * | * | * | * | * | * | * | * | * | * | * | * | * | * | * | * |
| **Gastrointestinal reaction** | * | * | * | * | 7 | 0 | 10 | 0 | * | * | * | * | * | * | * | * | * | * | * | * | * | * | * | * |
| **Plural efusion** | * | * | * | * | * | * | * | * | 5 | 0 | 3 | 0 | * | * | * | * | * | * | * | * | * | * | * | * |
| **Thyroid dysfunction** | * | * | * | * | * | * | * | * | * | * | * | * | * | * | * | * | 8 | 6 | 7 | 0 | * | * | * | * |
| **infection** | * | * | * | * | * | * | * | * | * | * | * | * | * | * | * | * | * | * | * | * | 1 | 0 | 1 | 1 |
| **Hepatic encephalopathy** | * | * | * | * | * | * | * | * | * | * | * | * | * | * | * | * | * | * | * | * | 0 | 0 | 1 | 1 |
| **Acute kidney injury** | * | * | * | * | * | * | * | * | * | * | * | * | * | * | * | * | * | * | * | * | 1 | 1 | 1 | 1 |
| IT-TKI: Interventional therapy and TKIs; *: not available. | | | | | | | | | | | | | | | | | | | | | | | | |

| **Supplementary Table 5**  **Total AEs and grade 3-4 adverse events of ICIs in the triple therapy group** | | | | | | | | | | | | |
| --- | --- | --- | --- | --- | --- | --- | --- | --- | --- | --- | --- | --- |
|  | **Lin 2023** | | **Xia 2023** | | **Yu 2023** | | **Zhang 2023** | | **Zou 2023** | | **Wu 2024** | |
|  | **total** | **G3-4** | **total** | **G3-4** | **total** | **G3-4** | **total** | **G3-4** | **total** | **G3-4** | **total** | **G3-4** |
| **RCCEP** | * | * | 1 | 0 | * | * | * | * | * | * | * | * |
| **Immunological Hypothyroidism** | * | * | 5 | 0 | 4 | 0 | 1 | 0 | * | * | 2 | 0 |
| **Immunological Hyperthyroidism** | * | * | 2 | 0 | * | * | * | * | * | * | * | * |
| **Immunological pneumonia** | * | * | 1 | 0 | * | * | 2 | 0 | * | * | * | * |
| **Suppurative tonsillitis** | * | * | * | * | 1 | 0 | * | * | * | * | * | * |
| **Intestinal infection** | * | * | * | * | 1 | 1 | * | * | * | * | * | * |
| **Immunological Hepatitis** | * | * | * | * | 1 | 1 | * | * | * | * | * | * |
| **Immunological rash** | * | * | * | * | 2 | 1 | * | * | * | * | * | * |
| **Immunological enteritis** | * | * | * | * | * | * | 1 | 1 | * | * | * | * |
| **Immunological myocarditis** | * | * | * | * | * | * | 1 | 1 | * | * | 1 | 0 |
| ICIs: immune checkpoint inhibitors; *:not available; RCCEP: reactive cutaneous capillary endothelial proliferation. | | | | | | | | | | | | |
